# Mentored Flip-Jigsaw method: enhancing sectional anatomy education

**DOI:** 10.1186/s12909-025-08129-z

**Published:** 2025-11-07

**Authors:** Khatere Roozbehi, Sajad Borzoueisileh, Marjan Roozbehi, Hassan Vafapour, Ali Aazami, Amrollah Roozbehi

**Affiliations:** 1 Medical School, Yasuj University of Medical Sciences, Yasuj, Iran; 2 Radiobiology and Radiation Protection (Biomedicine), Department of Radiation Sciences,, Yasuj University of Medical Sciences, Yasuj, Iran; 3 Cardiovascular Research Center, Rajaie Cardiovascular Institute, Tehran, Iran; 4 Present Address: Cellular and Molecular Research Center, Yasuj University of Medical Sciences, Yasuj, Iran; 5 Department of Radiology Sciences, School of Paramedical Sciences, Yasuj University of Medical Sciences, Yasuj, Iran; 6 Department of Anatomical Sciences, School of Medicine, Yasuj University of Medical Sciences, Yasuj, Iran

**Keywords:** Flipped classroom, Jigsaw, Mentorship, Peer learning

## Abstract

**Background:**

Student-centered teaching strategies are increasingly used in health sciences education to promote critical thinking and deeper learning. This study aimed to evaluate the effectiveness of combining the flipped Jigsaw method with structured mentorship, compared to the flipped Jigsaw method alone, in improving educational outcomes for radiologic technology students learning sectional anatomy.

**Methods:**

A quasi-experimental, two-phase study was conducted with 42 radiologic technology students across two academic periods. In the first phase, students were taught using the flipped Jigsaw method. In the second phase, the same instructional approach was enhanced with peer mentorship from senior students. The outcomes assessed included metacognition, learner satisfaction, interaction, learning performance, mental effort, and educational efficiency. Standardized questionnaires and anatomy tests were used for data collection. Statistical analyses included repeated measures ANOVA, with significance set at *p* < 0.05.

**Results:**

Students in the mentorship-enhanced group showed significantly higher scores in several metacognitive domains, particularly conditional knowledge (*p* = 0.015). Learner satisfaction improved across six of seven measured dimensions, including motivation (*p* = 0.006) and content (*p* = 0.029). Interaction skills increased significantly in all eight assessed areas, such as participation (*p* = 0.007) and communication (*p* < 0.001). Although learning scores increased, the difference was not statistically significant (*p* = 0.107). However, mental effort was significantly lower (*p* = 0.008), and educational efficiency was significantly higher (*p* = 0.005) in the mentorship group.

**Conclusions:**

Integrating structured mentorship into the flipped Jigsaw model significantly enhanced student outcomes in metacognition, satisfaction, and academic interaction while reducing mental effort and improving educational efficiency. This combined approach may offer a more effective instructional model for complex content such as sectional anatomy. Future research should explore its application in broader academic settings and assess long-term learning retention.

## Introduction

Traditional teacher-centered instruction, which often involves direct teaching and lecture-based delivery, continues to be widespread in educational settings and is effective for conveying standardized content [1, 2]. In this approach, the teacher is viewed as the primary source of knowledge, while students tend to play a passive role [3]. Although this model supports consistency and content delivery [4], it may limit students’ cognitive development, engagement, and ability to apply knowledge in clinical or real-life contexts [5]. In contrast, student-centered approaches, which emphasize active participation, problem-solving, and collaboration, have gained popularity for fostering critical thinking and learner autonomy [6–9]. These methods allow learners to take ownership of their educational journey and better adapt to evolving professional environments [3].

Student-centered teaching includes a variety of methods, such as flipped classrooms, project-based learning, and collaborative methods like Jigsaw [7, 10]. In flipped classrooms, students prepare by reviewing materials before class and engage in discussions and activities during class time, which enhances their application of knowledge and critical thinking skills [2, 11]. The Jigsaw method, a collaborative learning approach in which students become “experts” on specific subtopics and then teach their peers, has shown to improve communication skills, retention, and active participation [12–14]. When integrated, the flipped and Jigsaw methods form the “flip-Jigsaw” model, which combines the strengths of both approaches by maximizing pre-class preparation and peer-based learning in class [15, 16].

In recent years, mentorship has been identified as a powerful complement to both student-centered and teacher-centered approaches, particularly by offering personalized guidance and fostering professional academic skills, including professional development and education [17–19]. Mentorship also provides a platform for continuous feedback and role modeling, which aligns well with the goals of modern medical education and lifelong learning [20, 21]. Adding mentorship to the flip-Jigsaw model can further support students by enhancing motivation, satisfaction, metacognitive reflection, academic interaction and improving clinical reasoning skills [17, 22, 23].

The aim of this study is to design, implement and assess a teaching method for sectional anatomy that combines the flip-Jigsaw approach with structured mentorship, to enhance the educational experience and clinical preparedness of radiologic technology students.

## Methods

### Study design

This was a prospective, two-phase, quasi-experimental study conducted over two academic periods. The study employed a comparative design to assess the outcomes of two instructional interventions applied to enhance cognition and academic skills of radiologic technology students.

### Setting and participants

The study was conducted at Yasuj University of Medical Sciences (YUMS), Iran. The research sample included all radiologic technology students enrolled in two consecutive academic periods of spring 2023 and spring 2024 who participated in a sectional anatomy course in the fourth semester, in 3 units (51 h theoretical classes). The students passed limb, trunk and head and neck anatomy, each in two units (26 h theoretical and 17 h practical classes), in their previous three semesters. None of the students had prior exposure to the course content or the instructional methods used.

To assess the baseline knowledge of students in each class, we calculated their mean baseline scores out of a maximum of 20, based on their performance in anatomy courses taken prior to the start of our study. These scores reflect the average grades or assessment results from all relevant anatomy coursework completed by the students before participating in sectional anatomy course.

### Instructional interventions and outcomes

In class 1, the flipped Jigsaw method was implemented in the semester of spring 2023. Students were organized into “home” and “expert” groups to collaboratively learn and practice cross-sectional anatomy using models, atlases and sectional images. Students were stratified based on their prior performance in anatomy courses. Block randomization was then employed to ensure an equal distribution of high- and low-performing students across the study groups. Seven students were assigned to each group. The class was facilitated by one tutor and three mentors (one for each group). Prior to the start of the course, a needs assessment was conducted through interviews with the aim of evaluating the current situation and identifying educational needs from the perspectives of both mentors and students. The primary objective of this needs assessment was to examine the effectiveness of existing instructional methods, including student-centered approaches.

**Fig. 1:**
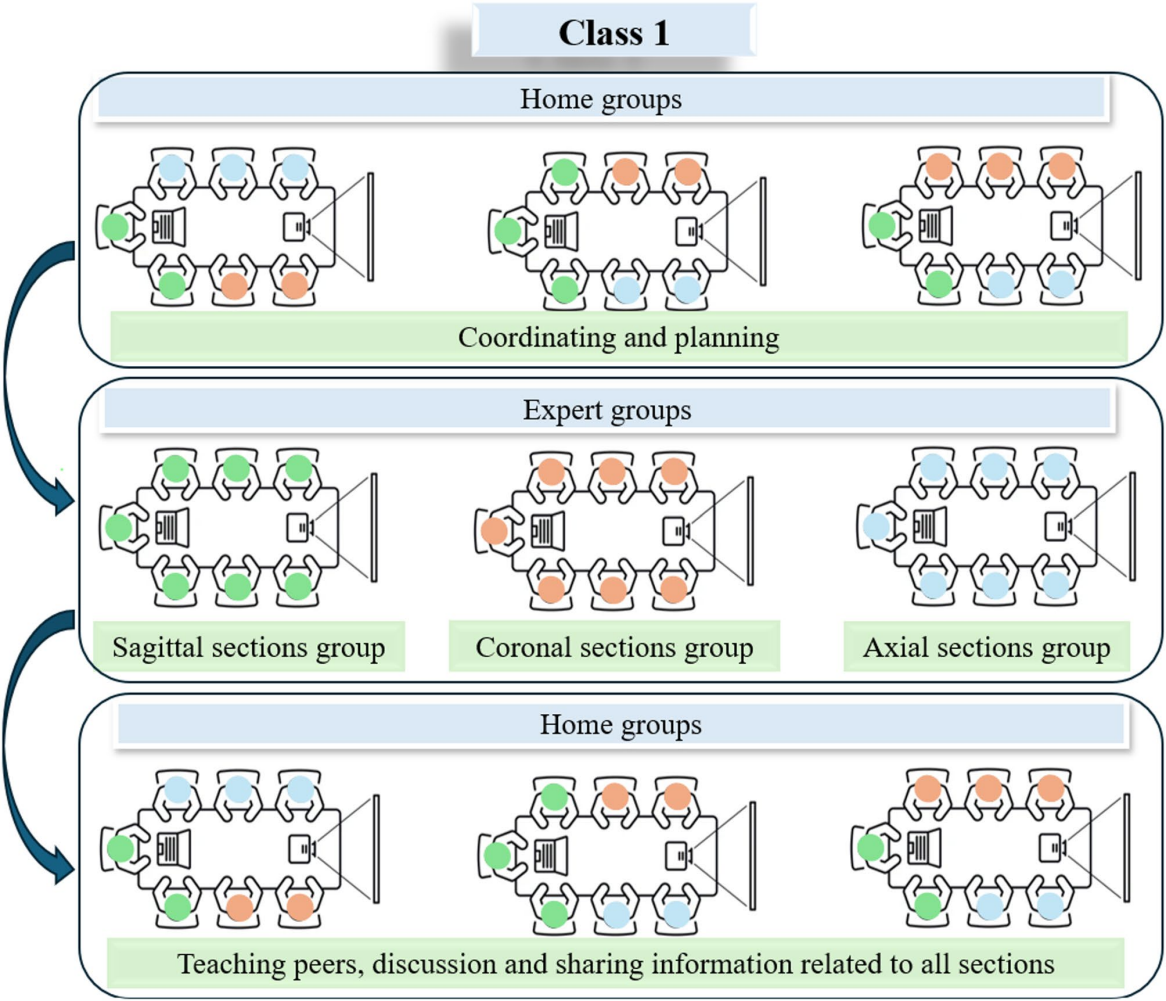
Flipped Jigsaw flow diagram in class 1

The content was divided into three main sections: head and neck (brain, face, and neck), trunk (spine, thorax, abdomen, and pelvis), and limbs (shoulder, elbow, wrist, pelvis, thigh, knee, and foot). In the first home group, each student was assigned one of the sagittal, coronal, or axial cuts, and then all students responsible for the same type of cut gathered in an expert group. In the expert groups, each subsection was thoroughly practiced using sectional images corresponding to the assigned cut (sagittal, coronal, or axial). In the second home group, the expert students took turns presenting their assigned content to the other members (Fig. 1). The sessions were held once a week, each lasting two hours.

**Fig. 2:**
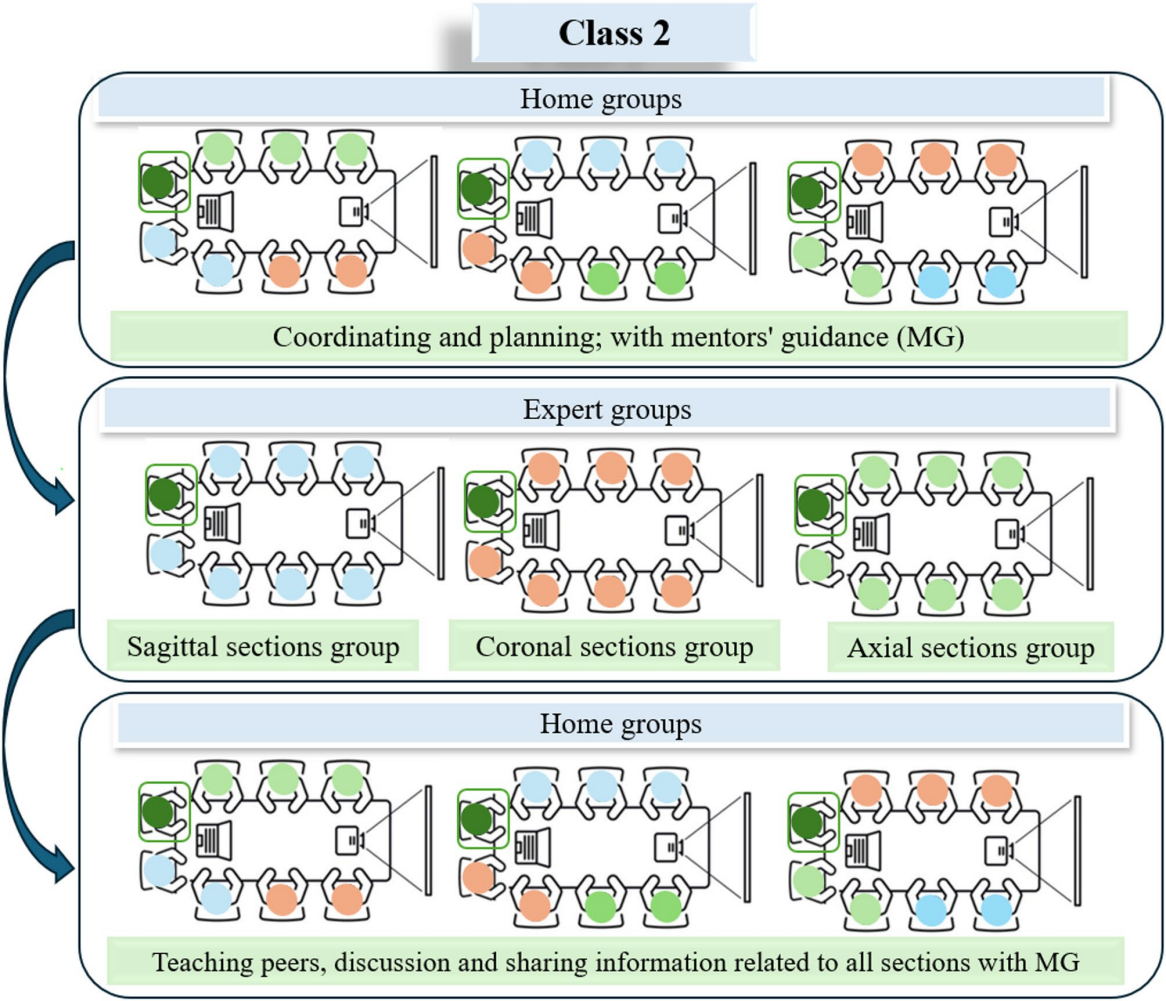
Flipped Jigsaw flow diagram in class 2

In class 2, the flipped Jigsaw method was supplemented with mentorship in the semester of spring 2024. All stages of group formation and content assignment to each student were carried out similarly to the previous class, but in consultation with senior students who served as mentors throughout the entire implementation and had a good understanding of the junior students, including their learning habits and preferences. Seven students were assigned to each group. Mentors, assigned to each group, facilitated academic interaction by providing motivation, guidance, technical troubleshooting, and immediate feedback to enhance educational efficiency. Both interventions utilized identical educational materials, including reference books and atlases, anatomical models, images, and instructor-prepared slides, structured as Reusable Learning Objects (RLOs). The content was divided into manageable sections, and students engaged in theoretical and practical sessions over two weeks. A dedicated tutor monitored group dynamics and provided feedback in both periods (Fig. 2).

To ensure readiness, mentors underwent prior training, and necessary infrastructure was established. A coordination session was held with the educational team, mentors, and students to simplify content delivery and foster student engagement. Mentors, due to their cognitive and social alignment with students, facilitated feedback exchange between students and instructors.

The study evaluated six key outcomes across two classes. The Metacognition Outcome included eight dimensions: Procedural, Declarative, Conditional, Information Management, Debugging, Planning, Comprehension, and Evaluation. The Satisfaction Outcome comprised seven dimensions: Objectives, Methods, Motivation, Content, Management, Aids, and Impacts. The Interaction Outcome consisted of eight dimensions: Relationship, Preparedness, Discussion, Participation, Share, Concern, Agreement, and Closure. Three other educational outcomes were investigated including Learning, Mental Effort, and Educational Efficiency.

### Data collection tools

Data was collected using a combination of standard questionnaires and anatomy tests. Learning outcome was assessed through formative and summative tests. Interaction skills were measured using the Kalamazoo Communication Skills Checklist. Metacognition was assessed with the Schraw and Dennison Metacognitive Awareness Inventory. Both questionnaires are provided in the Supplementary file. Mental effort was quantified using a 0–12 scoring system (0 = least effort, 12 = greatest effort) for each question. Educational efficiency was calculated based on the relationship between learning outcome and mental effort using Formula 1. Student satisfaction was evaluated through a learner satisfaction questionnaire.

**Formula 1.**

$$\:\text{E}\text{d}\text{u}\text{c}\text{a}\text{t}\text{i}\text{o}\text{n}\text{a}\text{l}\:\text{e}\text{f}\text{f}\text{i}\text{c}\text{i}\text{e}\text{n}\text{c}\text{y}=\:\frac{Z\:score\:of\:Learning\:-Z\:score\:of\:Mental\:effort\:}{\sqrt{2}}$$

The anatomy tests were image-based practical examinations conducted in the virtual examination hall and included multiple-choice questions as well as sectional image identification.

### Data analysis

Data was analyzed using SPSS software (version 25). Descriptive statistics were used for demographic and baseline data. For normally distributed data, repeated measures ANOVA was employed to compare outcomes between the two periods. For non-normally distributed data, equivalent non-parametric tests were used. Statistical significance was set at *p* < 0.05.

### Ethical considerations

Ethical approval was obtained from the institutional review board of Yasuj University of Medical Sciences. All students and mentors provided informed consent after receiving detailed information about the study’s purpose. Anonymity was maintained for all questionnaire responses.

## Results

### Baseline characteristics

In this study, we implemented the Jigsaw Method in Class and the Jigsaw Method combined with mentorship in Class 2, with each class consisting of 21 students. The mean age of Class 1 was 23.82 ± 0.98 years, and Class 2 had a mean age of 23.23 ± 1.47 years. The mean baseline score for previous anatomy courses was 12.72 ± 1.2 for Class 1 and 13.29 ± 0.9 for Class 2. A comparison of baseline anatomy knowledge between the two classes yielded a P-value of 0.082, indicating no significant difference in their prior anatomy knowledge.

### Needs assessment

The needs assessment conducted for sectional anatomy education revealed that traditional teaching methods fail to actively engage students and are predominantly characterized by one-way communication from instructors. Additionally, a noticeable deficiency was observed in the development of practical skills, particularly in interpreting cross-sectional images.

### Metacognition domains

Higher mean scores were seen in Class 2 across all metacognitive dimensions. The Conditional knowledge dimension reached statistical significance between two classes (*P* = 0.015), while other dimensions showed unsignificant increase from Class 1 to Class 2. The detailed results of this outcome are presented in Table 1.

**Table 1:** Metacognition outcome reported based on eight dimensions

Metacognition outcome	Class	N	Mean	Std. deviation	Mean difference	95% CI of the difference		*P*-Value
						Lower	Upper	
Procedural	1	18	3.57	0.97	−0.419	−0.946	0.109	0.116
	2	21	3.99	0.64				
Declarative	1	18	3.77	0.97	−0.279	−0.791	0.233	0.277
	2	21	4.05	0.58				
Conditional	1	18	3.49	0.99	−0.625	−1.120	−0.131	0.015
	2	21	4.11	0.49				
Information Management	1	18	3.70	0.79	−0.305	−0.736	0.127	0.161
	2	21	4.01	0.53				
Debugging	1	18	3.74	0.86	−0.351	−0.816	0.115	0.135
	2	21	4.10	0.56				
Planning	1	18	3.60	0.88	−0.337	−0.805	0.131	0.153
	2	21	3.93	0.54				
Comprehension	1	18	3.71	0.96	−0.259	−0.755	0.238	0.298
	2	21	3.97	0.54				
Evaluation	1	18	3.57	0.91	−0.474	−0.967	0.020	0.059
	2	21	4.05	0.59				

### Satisfaction domains

Class 2 demonstrated greater mean scores in all satisfaction dimensions. Statistically significant differences were observed between the two classes for Objectives (*P* = 0.045), Methods (*P* = 0.012), Motivation (*P* = 0.006), Content (*P* = 0.029), Management (*P* = 0.004), and Impacts (*P* = 0.047), while the Aids dimension showed a non-significant increase from Class 1 to Class 2 (*P* = 0.058). The detailed results of this outcome are presented in Table 2.

**Table 2:** Satisfaction outcome reported based on seven dimensions

Satisfaction Outcome	Class	N	Mean	Std. deviation	Mean difference	95% CI of the difference		*P*-Value
						Lower	Upper	
Objectives	1	21	3.95	0.86	−0.477	−0.943	−0.012	0.045
	2	21	4.43	0.66				
Methods	1	21	3.55	1.18	−0.750	−1.329	−0.171	0.012
	2	21	4.30	0.65				
Motivation	1	21	3.80	0.95	−0.659	−1.122	−0.196	0.006
	2	21	4.45	0.51				
Content	1	21	3.68	1.11	−0.614	−1.162	−0.065	0.029
	2	21	4.30	0.63				
Management	1	21	3.73	1.21	−0.841	−1.404	−0.277	0.004
	2	21	4.57	0.50				
Aids	1	21	4.05	1.05	−0.500	−1.018	0.018	0.058
	2	21	4.55	0.60				
Impacts	1	21	3.95	0.79	−0.409	−0.813	−0.005	0.047
	2	21	4.36	0.51				

### Interaction domains

Higher average scores were achieved by Class 2 across all interaction-related dimensions. Statistically significant differences were found between the two classes for Relationship (*P* = 0.003), Preparedness (*P* = 0.002), Discussion (*P* = 0.001), Participation (*P* = 0.007), Share (*P* < 0.001), Concern (*P* < 0.001), Agreement (*P* < 0.001), and Closure (*P* = 0.005). The detailed results of this outcome are presented in Table 3.

**Table 3:** Interaction outcome reported based on eight dimensions

Interaction outcome	Class	N	Mean	Std. deviation	Mean difference	95% CI of the difference		*P*-Value
						Lower	Upper	
Relationship	1	19	3.75	0.89	−0.682	−1.118	−0.245	0.003
	2	21	4.43	0.46				
Preparedness	1	19	3.62	0.68	−0.585	−0.947	−0.223	0.002
	2	21	4.20	0.46				
Discussion	1	19	3.69	0.86	−0.733	−1.148	−0.317	0.001
	2	21	4.43	0.41				
Participation	1	19	3.82	0.74	−0.548	−0.933	−0.162	0.007
	2	21	4.36	0.46				
Share	1	19	3.45	0.93	−0.939	−1.386	−0.492	0.000
	2	21	4.39	0.43				
Concern	1	19	3.57	0.79	−0.900	−1.301	−0.499	0.000
	2	21	4.47	0.45				
Agreement	1	19	3.66	0.78	−0.797	−1.186	−0.407	0.000
	2	21	4.45	0.43				
Closure	1	19	3.83	0.75	−0.614	−1.029	−0.199	0.005
	2	21	4.44	0.56				

### Learning, mental effort and educational efficiency outcomes

Class 2 showed increased mean scores for Learning and Educational Efficiency, with a reduced mean score for Mental Effort. Statistically significant differences were found between the two classes for Mental Effort (*P* = 0.008) and Educational Efficiency (*P* = 0.005), whereas the Learning dimension showed a non-significant increase from Class 1 to Class 2 (*P* = 0.107). Table 4 shows the detailed results of these outcomes.

**Table 4:** Learning, mental effort and educational efficiency outcomes compared between class 1 and class 2

Educational outcome	Class	N	Mean	Std. deviation	Mean difference	95% CI of the difference		*P*-Value
						Lower	Upper	
Learning	1	21	76.77	13.02	−5.273	−11.74	1.194	0.107
	2	21	82.04	7.50				
Mental Effort	1	21	6.876	1.21	0.951	0.2626	1.6385	0.008
	2	21	5.925	1.14				
Educational Efficiency	1	21	−0.451	1.20	−0.900	−1.507	−0.294	0.005
	2	21	0.449	0.74				

## Discussion

**Fig. 3:**
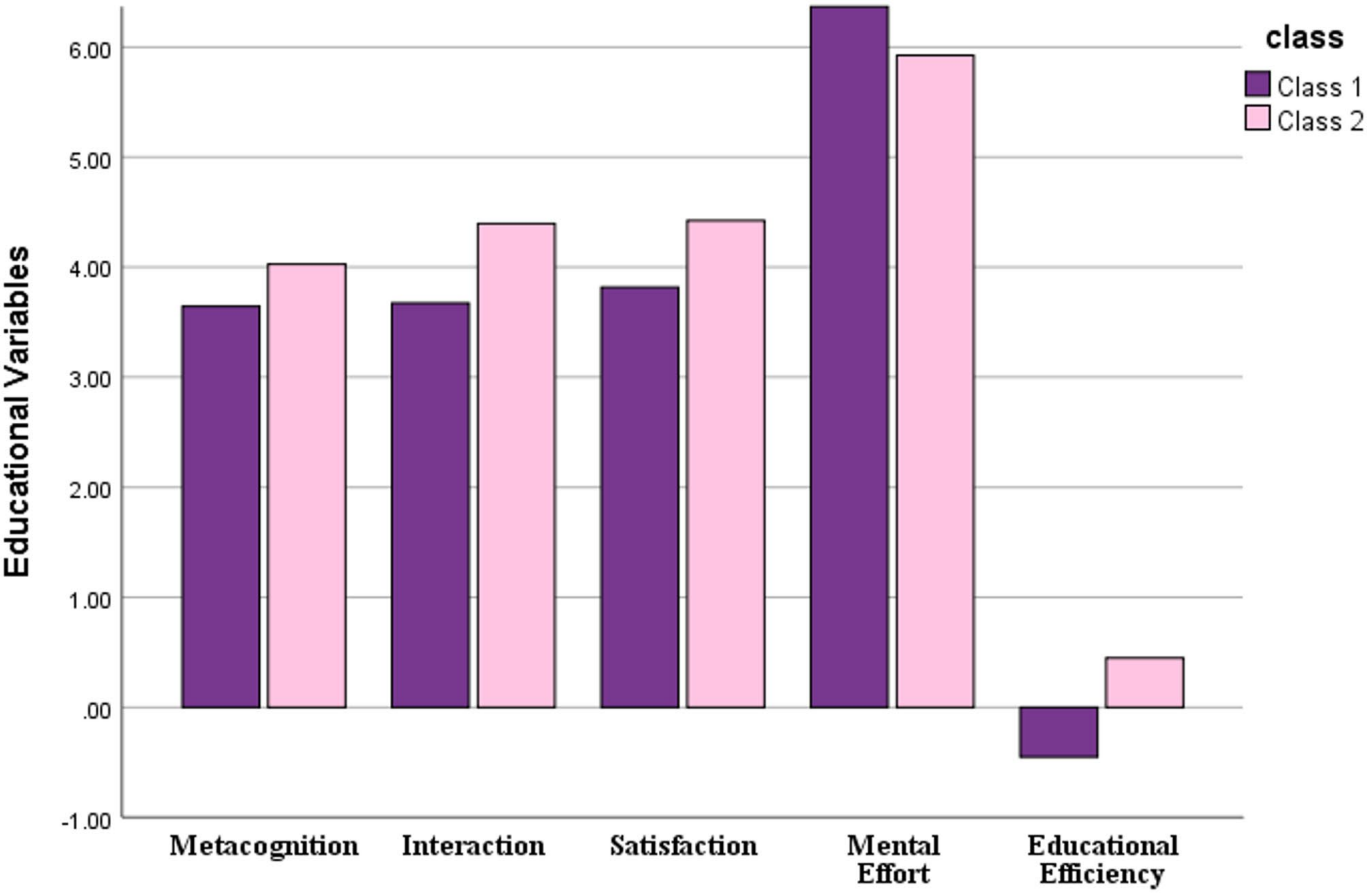
Bar plot demonstrating a comparsion of mean outcomes between class 1 and class 2

This study evaluated the effectiveness of a student-centered teaching method combining the flipped Jigsaw approach with mentorship compared to the flipped Jigsaw method alone in teaching sectional anatomy to radiologic technology students. The findings of our needs assessment emphasize the need to revise current teaching methods and adopt innovative, student-centered approaches to improve learning and professional readiness. Our results demonstrated that integrating mentorship with the flipped Jigsaw method significantly improved outcomes across metacognition, satisfaction, interaction, learning and educational efficiency while reducing mental effort. A bar plot demonstrating a comparsion of mean outcomes between class 1 and class 2 is seen in Fig. 3. These findings align with the growing body of evidence supporting student-centered approaches supplemented by mentorship in medical education.

The metacognition outcomes revealed higher mean scores across all dimensions in Class 2, with a statistically significant improvement in conditional knowledge (*P* = 0.015). This suggests that mentorship enhances students’ ability to apply knowledge strategically in context. The non-significant improvements in other dimensions, such as procedural and declarative knowledge, indicate a positive trend that may require larger sample sizes to achieve statistical significance. These results are consistent with Sanaei et al. (2019), who found that the Jigsaw method improved self-regulated learning in nursing students, suggesting that collaborative methods foster metacognitive skills [24]. However, our study incorporated mentorship, which likely amplified the effect on conditional knowledge by providing personalized guidance, aligning with Caruso et al. [25], who reported mentorship’s role in enhancing professional development [25].

After adding mentorship, higher satisfaction scores were seen across six of seven dimensions, including objectives (*P* = 0.045), methods (*P* = 0.012), motivation (*P* = 0.006), content (*P* = 0.029), management (*P* = 0.004), and impacts (*P* = 0.047). These findings indicate that mentorship enhances students’ perceptions of the learning experience, likely due to personalized feedback and role modeling. Our study extends their findings by demonstrating that mentorship further amplifies satisfaction across multiple dimensions, possibly by fostering a supportive learning environment. However, the non-significant increase in the aids dimension (*P* = 0.058) suggests that both groups perceived learning resources similarly, consistent with Bakr et al.’s (2012–2016) observation that digital resources alone may not fully account for improved outcomes without active engagement strategies [26].

Interaction outcomes showed significant improvements in all eight dimensions for Class 2, including relationship (*P* = 0.003), preparedness (*P* = 0.002), discussion (*P* = 0.001), participation (*P* = 0.007), share (*P* < 0.001), concern (*P* < 0.001), agreement (*P* < 0.001), and closure (*P* = 0.005). These results highlight mentorship’s role in fostering collaborative and communicative skills. This corroborates Wilson et al. [27], who found that the Jigsaw method improved communication and collaborative learning skills in pharmaceutical management students, though their study did not include mentorship [27]. The significant improvements in our study suggest that mentorship enhances peer interactions by providing structured guidance, aligning with Caruso et al. [25], who noted mentorship’s positive impact on academic interactions [25]. The robust interaction outcomes in Class 2 also echo Amani et al. (2024), who identified enhanced social skills as a key benefit of the Jigsaw method, further amplified by mentorship in our study [28].

Integrating flipped Jigsaw with mentorship demonstrated a non-significant increase in learning scores (*P* = 0.107), a significant reduction in mental effort (*P* = 0.008), and a significant improvement in educational efficiency (*P* = 0.005). The reduced mental effort in Class 2 suggests that mentorship alleviates cognitive load, possibly by clarifying complex concepts through individualized support. This aligns with Gillispie et al. (2015), who reported improved student performance in a flipped classroom setting, though their study did not assess mental effort [2]. The significant improvement in educational efficiency in our study indicates that mentorship optimizes learning outcomes relative to cognitive demands, a finding not explicitly addressed in prior studies like Lin et al. [7], who noted enhanced problem-solving and critical thinking in flipped classroom settings [7].

Some challenges in the implementation of the Jigsaw learning method, are excessive time consumption, inadequate logistics, limited faculty supervision, and insufficient preparation by peers [29]. To overcome these, we optimized the duration of activities to prevent fatigue, managed group allocation and movement to minimize noise and confusion, ensured more frequent faculty monitoring and guidance, and encouraged thorough preparation through pre-session reading materials and clearer role assignments.

## Conclusion

The integration of mentorship with the flipped Jigsaw method significantly enhances metacognition, satisfaction, interaction, and educational efficiency while reducing mental effort in radiologic technology students learning sectional anatomy. This study underscores the potential of integrating mentorship with active learning strategies to prepare radiologic technology students for clinical practice effectively.

## Study limitations

Limitations include the relatively small sample size, which may limit the generalizability of the findings, and the quasi-experimental, single-site design led by a single instructor, which introduces the possibility of cohort-specific or novelty effects. Additionally, the absence of a delayed post-test prevents assessment of long-term knowledge retention, and the lack of extended follow-up restricts evaluation of whether gains translate into clinical application. Future research should address these issues by employing larger, multi-site cohorts, including multiple instructors, and incorporating long-term follow-up measures to confirm the durability and practical impact of these benefits.

## Data Availability

The data supporting the findings of this study are available upon request.
